# Chlorido{4-chloro-2-[(2-morpholinoeth­yl)imino­meth­yl]phenolato-κ^3^ *N*,*N*′,*O*}copper(II)

**DOI:** 10.1107/S1600536809025215

**Published:** 2009-07-04

**Authors:** Nurul Azimah Ikmal Hisham, Hapipah Mohd Ali, Seik Weng Ng

**Affiliations:** a Department of Chemistry, University of Malaya, 50603 Kuala Lumpur, Malaysia

## Abstract

The Cu^II^ atom in the title compound, [Cu(C_13_H_16_ClN_2_O_2_)Cl], exists in a distorted square-planar coordination environment as the deprotonated Schiff base chelates to the Cu^II^ atom through three atom sites. In the crystal structure, adjacent mol­ecules are linked by a Cu⋯Cl [3.011 (1) Å] bridge, generating a linear chain running along the *b* axis of the ortho­rhom­bic unit cell.

## Related literature

A similar deprotonated Schiff base is bidentate in bis­{5-meth­oxy-2-[(2-morpholinoeth­yl)imino­meth­yl]phenolato}nickel; see: Mohd Lair *et al.* (2009 ▶).
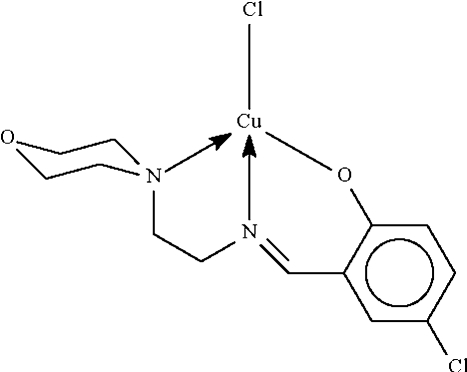


## Experimental

### 

#### Crystal data

[Cu(C_13_H_16_ClN_2_O_2_)Cl]
*M* *_r_* = 366.72
Orthorhombic, 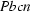

*a* = 23.0936 (6) Å
*b* = 8.4890 (2) Å
*c* = 14.0582 (3) Å
*V* = 2756.0 (1) Å^3^
*Z* = 8
Mo *K*α radiation
μ = 1.97 mm^−1^
*T* = 140 K
0.40 × 0.10 × 0.02 mm

#### Data collection

Bruker SMART APEX diffractometer
Absorption correction: multi-scan (*SADABS*; Sheldrick, 1996 ▶) *T*_min_ = 0.506, *T*_max_ = 0.962
17248 measured reflections
3156 independent reflections
2416 reflections with *I* > 2σ(*I*)
*R*_int_ = 0.050

#### Refinement

*R*[*F* ^2^ > 2σ(*F* ^2^)] = 0.042
*wR*(*F* ^2^) = 0.113
*S* = 1.09
3156 reflections
181 parameters
H-atom parameters constrained
Δρ_max_ = 0.76 e Å^−3^
Δρ_min_ = −0.84 e Å^−3^

### 

Data collection: *APEX2* (Bruker, 2008 ▶); cell refinement: *SAINT* (Bruker, 2008 ▶); data reduction: *SAINT*; program(s) used to solve structure: *SHELXS97* (Sheldrick, 2008 ▶); program(s) used to refine structure: *SHELXL97* (Sheldrick, 2008 ▶); molecular graphics: *X-SEED* (Barbour, 2001 ▶); software used to prepare material for publication: *publCIF* (Westrip, 2009 ▶).

## Supplementary Material

Crystal structure: contains datablocks global, I. DOI: 10.1107/S1600536809025215/xu2547sup1.cif

Structure factors: contains datablocks I. DOI: 10.1107/S1600536809025215/xu2547Isup2.hkl

Additional supplementary materials:  crystallographic information; 3D view; checkCIF report

## Figures and Tables

**Table 1:** Selected bond lengths (Å)

Cu1—O1	1.907 (2)
Cu1—N1	1.947 (3)
Cu1—N2	2.105 (3)
Cu1—Cl1	2.2620 (9)

**Fig. 1.**
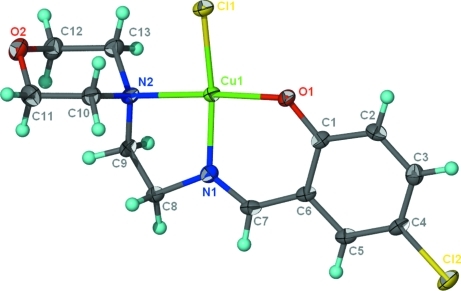
Thermal ellipsoid plot (Barbour, 2001) of the CuCl(C13H16ClN2O2) at the 70% probability level. Hydrogen atoms are drawn as spheres of arbitrary radius.

## supplementary crystallographic information

### Experimental

The Schiff base was synthesized by condensing N-2-(aminoethyl)morpholine (0.80 g, 6.1 mmol) and 5-chlorosalicylaldehyde (0.96 g, 6.1 mmol) in ethanol; the reactants were heated for 2 hours. Copper(II) chloride (1.00 g, 6.1 mmol) was added and the heating continued for another 5 hour. The solvent was removed and the product recrystallized from methanol.

### Refinement

Hydrogen atoms were placed at calculated positions (C–H 0.95–0.99 Å) and were treated as riding on their parent carbon atoms, with *U*(H) set to 1.2 times *U*_eq_(*C*).

### Crystal data

**Table:**

[Cu(C_13_H_16_ClN_2_O_2_)Cl]	*F*(000) = 1496
*M**_r_* = 366.72	*D*_x_ = 1.768 Mg m^−^^3^
Orthorhombic, *P**b**c**n*	Mo *K*α radiation, λ = 0.71073 Å
Hall symbol: -P 2n 2ab	Cell parameters from 4043 reflections
*a* = 23.0936 (6) Å	θ = 2.6–28.1°
*b* = 8.4890 (2) Å	µ = 1.97 mm^−^^1^
*c* = 14.0582 (3) Å	*T* = 140 K
*V* = 2756.0 (1) Å^3^	Plate, green
*Z* = 8	0.40 × 0.10 × 0.02 mm

### Data collection

**Table:**

Bruker SMART APEX diffractometer	3156 independent reflections
Radiation source: fine-focus sealed tube	2416 reflections with *I* > 2σ(*I*)
graphite	*R*_int_ = 0.050
ω scans	θ_max_ = 27.5°, θ_min_ = 1.8°
Absorption correction: multi-scan (*SADABS*; Sheldrick, 1996)	*h* = −29→30
*T*_min_ = 0.506, *T*_max_ = 0.962	*k* = −10→10
17248 measured reflections	*l* = −18→18

### Refinement

**Table:**

Refinement on *F*^2^	Primary atom site location: structure-invariant direct methods
Least-squares matrix: full	Secondary atom site location: difference Fourier map
*R*[*F*^2^ > 2σ(*F*^2^)] = 0.042	Hydrogen site location: inferred from neighbouring sites
*wR*(*F*^2^) = 0.113	H-atom parameters constrained
*S* = 1.09	*w* = 1/[σ^2^(*F*_o_^2^) + (0.0476*P*)^2^ + 6.7938*P*] where *P* = (*F*_o_^2^ + 2*F*_c_^2^)/3
3156 reflections	(Δ/σ)_max_ = 0.001
181 parameters	Δρ_max_ = 0.76 e Å^−^^3^
0 restraints	Δρ_min_ = −0.84 e Å^−^^3^

### Fractional atomic coordinates and isotropic or equivalent isotropic displacement parameters (Å^2^)

**Table:**

	*x*	*y*	*z*	*U*_iso_*/*U*_eq_	
Cu1	0.229938 (18)	0.65838 (5)	0.49762 (3)	0.01385 (13)	
Cl1	0.24525 (4)	0.85643 (10)	0.39295 (6)	0.0181 (2)	
Cl2	0.43295 (4)	0.32750 (12)	0.83746 (7)	0.0253 (2)	
O1	0.30890 (10)	0.6693 (3)	0.53670 (17)	0.0159 (5)	
O2	0.03792 (12)	0.7569 (3)	0.36877 (18)	0.0225 (6)	
N1	0.20874 (13)	0.5357 (3)	0.60938 (19)	0.0141 (6)	
N2	0.14182 (13)	0.6347 (3)	0.46204 (19)	0.0131 (6)	
C1	0.33436 (15)	0.5909 (4)	0.6049 (2)	0.0131 (7)	
C2	0.39494 (16)	0.6087 (4)	0.6170 (2)	0.0158 (7)	
H2	0.4156	0.6769	0.5756	0.019*	
C3	0.42459 (16)	0.5294 (4)	0.6873 (2)	0.0157 (7)	
H3	0.4652	0.5438	0.6942	0.019*	
C4	0.39495 (16)	0.4279 (4)	0.7485 (2)	0.0169 (7)	
C5	0.33647 (16)	0.4082 (4)	0.7404 (2)	0.0158 (7)	
H5	0.3168	0.3396	0.7829	0.019*	
C6	0.30502 (15)	0.4889 (4)	0.6696 (2)	0.0139 (7)	
C7	0.24355 (16)	0.4685 (4)	0.6681 (2)	0.0149 (7)	
H7	0.2271	0.3998	0.7141	0.018*	
C8	0.14665 (15)	0.5035 (4)	0.6176 (2)	0.0147 (7)	
H8A	0.1272	0.5891	0.6531	0.018*	
H8B	0.1401	0.4030	0.6517	0.018*	
C9	0.12305 (15)	0.4933 (4)	0.5171 (2)	0.0147 (7)	
H9A	0.1376	0.3966	0.4859	0.018*	
H9B	0.0802	0.4882	0.5189	0.018*	
C10	0.11098 (16)	0.7815 (4)	0.4921 (2)	0.0168 (7)	
H10A	0.1293	0.8732	0.4607	0.020*	
H10B	0.1155	0.7949	0.5617	0.020*	
C11	0.04724 (17)	0.7799 (5)	0.4681 (3)	0.0209 (8)	
H11A	0.0280	0.6943	0.5040	0.025*	
H11B	0.0296	0.8810	0.4879	0.025*	
C12	0.06457 (16)	0.6146 (5)	0.3373 (3)	0.0202 (8)	
H12A	0.0585	0.6029	0.2679	0.024*	
H12B	0.0459	0.5239	0.3693	0.024*	
C13	0.12914 (16)	0.6129 (5)	0.3585 (2)	0.0173 (7)	
H13A	0.1457	0.5114	0.3371	0.021*	
H13B	0.1482	0.6981	0.3220	0.021*	

### Atomic displacement parameters (Å^2^)

**Table:**

	*U*^11^	*U*^22^	*U*^33^	*U*^12^	*U*^13^	*U*^23^
Cu1	0.0132 (2)	0.0170 (2)	0.0113 (2)	−0.00069 (18)	−0.00083 (15)	0.00270 (17)
Cl1	0.0202 (4)	0.0186 (4)	0.0154 (4)	−0.0010 (4)	−0.0009 (3)	0.0058 (3)
Cl2	0.0222 (5)	0.0295 (5)	0.0243 (5)	−0.0005 (4)	−0.0077 (4)	0.0126 (4)
O1	0.0146 (13)	0.0158 (13)	0.0174 (11)	0.0000 (10)	−0.0020 (10)	0.0069 (10)
O2	0.0209 (14)	0.0274 (15)	0.0193 (13)	0.0077 (12)	−0.0033 (11)	0.0008 (11)
N1	0.0152 (15)	0.0145 (15)	0.0126 (13)	−0.0010 (12)	−0.0018 (11)	−0.0015 (11)
N2	0.0163 (15)	0.0126 (14)	0.0103 (12)	0.0011 (12)	−0.0014 (11)	−0.0013 (11)
C1	0.0171 (18)	0.0117 (17)	0.0106 (15)	0.0007 (14)	−0.0021 (13)	−0.0006 (12)
C2	0.0173 (19)	0.0149 (17)	0.0151 (16)	−0.0030 (15)	0.0029 (13)	−0.0001 (13)
C3	0.0154 (18)	0.0144 (18)	0.0173 (16)	−0.0016 (14)	−0.0018 (13)	−0.0031 (13)
C4	0.0221 (19)	0.0159 (18)	0.0126 (15)	0.0024 (15)	−0.0035 (14)	0.0028 (13)
C5	0.0225 (19)	0.0127 (18)	0.0121 (15)	0.0002 (15)	0.0005 (13)	0.0006 (13)
C6	0.0191 (18)	0.0119 (16)	0.0106 (15)	0.0005 (14)	0.0001 (13)	−0.0007 (12)
C7	0.0205 (19)	0.0142 (17)	0.0100 (14)	0.0009 (14)	0.0029 (13)	−0.0002 (13)
C8	0.0140 (17)	0.0166 (17)	0.0133 (15)	−0.0007 (14)	−0.0002 (13)	0.0032 (13)
C9	0.0146 (17)	0.0161 (17)	0.0135 (16)	0.0003 (14)	−0.0030 (12)	0.0022 (13)
C10	0.0194 (18)	0.0146 (17)	0.0164 (16)	0.0035 (15)	0.0008 (14)	−0.0009 (14)
C11	0.022 (2)	0.0218 (19)	0.0191 (17)	0.0042 (17)	0.0028 (15)	−0.0031 (15)
C12	0.020 (2)	0.0238 (19)	0.0171 (17)	0.0009 (16)	−0.0031 (14)	−0.0020 (15)
C13	0.0191 (19)	0.0198 (18)	0.0130 (15)	0.0019 (15)	−0.0011 (13)	−0.0012 (14)

### Geometric parameters (Å, °)

**Table:**

Cu1—O1	1.907 (2)	C4—C5	1.366 (5)
Cu1—N1	1.947 (3)	C5—C6	1.410 (5)
Cu1—N2	2.105 (3)	C5—H5	0.9500
Cu1—Cl1	2.2620 (9)	C6—C7	1.430 (5)
Cu1—Cl1^i^	3.0107 (10)	C7—H7	0.9500
Cl2—C4	1.750 (3)	C8—C9	1.516 (4)
O1—C1	1.307 (4)	C8—H8A	0.9900
O2—C11	1.427 (4)	C8—H8B	0.9900
O2—C12	1.426 (5)	C9—H9A	0.9900
N1—C7	1.285 (4)	C9—H9B	0.9900
N1—C8	1.464 (4)	C10—C11	1.510 (5)
N2—C9	1.492 (4)	C10—H10A	0.9900
N2—C13	1.496 (4)	C10—H10B	0.9900
N2—C10	1.497 (4)	C11—H11A	0.9900
C1—C2	1.417 (5)	C11—H11B	0.9900
C1—C6	1.427 (5)	C12—C13	1.521 (5)
C2—C3	1.378 (5)	C12—H12A	0.9900
C2—H2	0.9500	C12—H12B	0.9900
C3—C4	1.396 (5)	C13—H13A	0.9900
C3—H3	0.9500	C13—H13B	0.9900
			
O1—Cu1—N1	91.95 (11)	N1—C7—C6	125.2 (3)
O1—Cu1—N2	176.00 (11)	N1—C7—H7	117.4
N1—Cu1—N2	84.12 (12)	C6—C7—H7	117.4
O1—Cu1—Cl1	90.10 (7)	N1—C8—C9	106.8 (3)
N1—Cu1—Cl1	164.06 (9)	N1—C8—H8A	110.4
N2—Cu1—Cl1	93.88 (8)	C9—C8—H8A	110.4
O1—Cu1—Cl1^i^	90.02 (8)	N1—C8—H8B	110.4
N1—Cu1—Cl1^i^	89.25 (9)	C9—C8—H8B	110.4
N2—Cu1—Cl1^i^	89.22 (8)	H8A—C8—H8B	108.6
Cl1—Cu1—Cl1^i^	106.56 (3)	N2—C9—C8	109.5 (3)
C1—O1—Cu1	128.1 (2)	N2—C9—H9A	109.8
C11—O2—C12	110.8 (3)	C8—C9—H9A	109.8
C7—N1—C8	118.6 (3)	N2—C9—H9B	109.8
C7—N1—Cu1	126.7 (3)	C8—C9—H9B	109.8
C8—N1—Cu1	114.2 (2)	H9A—C9—H9B	108.2
C9—N2—C13	110.4 (3)	N2—C10—C11	113.1 (3)
C9—N2—C10	112.6 (3)	N2—C10—H10A	109.0
C13—N2—C10	106.5 (3)	C11—C10—H10A	109.0
C9—N2—Cu1	103.6 (2)	N2—C10—H10B	109.0
C13—N2—Cu1	115.6 (2)	C11—C10—H10B	109.0
C10—N2—Cu1	108.2 (2)	H10A—C10—H10B	107.8
O1—C1—C2	118.5 (3)	O2—C11—C10	111.5 (3)
O1—C1—C6	124.3 (3)	O2—C11—H11A	109.3
C2—C1—C6	117.2 (3)	C10—C11—H11A	109.3
C3—C2—C1	121.6 (3)	O2—C11—H11B	109.3
C3—C2—H2	119.2	C10—C11—H11B	109.3
C1—C2—H2	119.2	H11A—C11—H11B	108.0
C2—C3—C4	120.0 (3)	O2—C12—C13	111.7 (3)
C2—C3—H3	120.0	O2—C12—H12A	109.3
C4—C3—H3	120.0	C13—C12—H12A	109.3
C5—C4—C3	120.6 (3)	O2—C12—H12B	109.3
C5—C4—Cl2	119.7 (3)	C13—C12—H12B	109.3
C3—C4—Cl2	119.7 (3)	H12A—C12—H12B	107.9
C4—C5—C6	120.5 (3)	N2—C13—C12	112.4 (3)
C4—C5—H5	119.7	N2—C13—H13A	109.1
C6—C5—H5	119.7	C12—C13—H13A	109.1
C5—C6—C7	117.6 (3)	N2—C13—H13B	109.1
C5—C6—C1	120.0 (3)	C12—C13—H13B	109.1
C7—C6—C1	122.4 (3)	H13A—C13—H13B	107.8
			
N1—Cu1—O1—C1	11.0 (3)	Cl2—C4—C5—C6	179.6 (3)
Cl1—Cu1—O1—C1	175.2 (3)	C4—C5—C6—C7	−177.2 (3)
Cl1^i^—Cu1—O1—C1	−78.2 (3)	C4—C5—C6—C1	0.6 (5)
O1—Cu1—N1—C7	−12.6 (3)	O1—C1—C6—C5	179.4 (3)
N2—Cu1—N1—C7	166.6 (3)	C2—C1—C6—C5	−1.3 (5)
Cl1—Cu1—N1—C7	−109.8 (4)	O1—C1—C6—C7	−2.9 (5)
Cl1^i^—Cu1—N1—C7	77.4 (3)	C2—C1—C6—C7	176.3 (3)
O1—Cu1—N1—C8	175.5 (2)	C8—N1—C7—C6	−179.5 (3)
N2—Cu1—N1—C8	−5.2 (2)	Cu1—N1—C7—C6	9.0 (5)
Cl1—Cu1—N1—C8	78.3 (4)	C5—C6—C7—N1	178.7 (3)
Cl1^i^—Cu1—N1—C8	−94.5 (2)	C1—C6—C7—N1	1.0 (5)
N1—Cu1—N2—C9	−21.8 (2)	C7—N1—C8—C9	−141.6 (3)
Cl1—Cu1—N2—C9	174.03 (19)	Cu1—N1—C8—C9	30.9 (3)
Cl1^i^—Cu1—N2—C9	67.48 (19)	C13—N2—C9—C8	169.0 (3)
N1—Cu1—N2—C13	−142.8 (3)	C10—N2—C9—C8	−72.1 (3)
Cl1—Cu1—N2—C13	53.1 (2)	Cu1—N2—C9—C8	44.6 (3)
Cl1^i^—Cu1—N2—C13	−53.4 (2)	N1—C8—C9—N2	−50.7 (4)
N1—Cu1—N2—C10	97.9 (2)	C9—N2—C10—C11	−67.4 (4)
Cl1—Cu1—N2—C10	−66.2 (2)	C13—N2—C10—C11	53.8 (4)
Cl1^i^—Cu1—N2—C10	−172.8 (2)	Cu1—N2—C10—C11	178.7 (2)
Cu1—O1—C1—C2	175.2 (2)	C12—O2—C11—C10	57.0 (4)
Cu1—O1—C1—C6	−5.6 (5)	N2—C10—C11—O2	−57.2 (4)
O1—C1—C2—C3	−179.9 (3)	C11—O2—C12—C13	−57.2 (4)
C6—C1—C2—C3	0.8 (5)	C9—N2—C13—C12	69.1 (4)
C1—C2—C3—C4	0.4 (5)	C10—N2—C13—C12	−53.5 (4)
C2—C3—C4—C5	−1.2 (5)	Cu1—N2—C13—C12	−173.8 (2)
C2—C3—C4—Cl2	179.9 (3)	O2—C12—C13—N2	57.4 (4)
C3—C4—C5—C6	0.7 (5)		

Symmetry codes: (i) −*x*+1/2, *y*−1/2, *z*.
